# The Hospital. Nursing Mirror

**Published:** 1898-09-17

**Authors:** 


### "The Hospital" Nursing Mirror.

Being the Nursing Section of "The Hospital."

[Contributions for this Section of "The Hospital" should be addressed to the Editor, The Hospital, 28 & 29, Southampton Street, Strand, London, W.C., and should have the word "Nursing" plainly written in left-hand top corner of the envelope.]

### News from the Nursing World.

### A ROYAL TRAGEDY.

The dastardly outrage on the Empress of Austria evokes a storm of righteous indignation against the monsters who avenge a sense of their own injuries, real or imaginary, in revolting crimes against the innocent. The noble lady, whose death from an assassin's stiletto fills everyone with horror, has been a central figure in romance and tragedy. In romance, when as a young girl the Emperor Francis Joseph of Austria, visiting her father's home to woo her sister fell in love with her; and later, when, after her marriage, she defied the etiquette of her Court, and ate, drank, and wore what she liked, and not what custom demanded; and again when, later still, her delight in outdoor sports and horsemanship led her into endless adventures. In tragedy, when suffering and broken-hearted, she lost her only son under circumstances too painful needlessly to mention, and as year by year ever since she has withdrawn to mourn her dead in seclusion. She was a good and charming woman, a loving and cherished wife, a devoted mother, and the beloved Empress of a loyal people, yet she has been the victim of as cruel an assassination as has ever been recorded. To the Emperor, her kinsfolk, and her people we offer our respectful sympathy.

### AMERICAN ARMY SISTERS.—PROPOSED TRAINING SCHOOLS.

With the growth of martial spirit and methods in America comes the necessity of providing for the health and healing of the soldier. From a force of 10,000 men, the standing army will probably be increased to 100,000; and, although America may not have to face the perennial crop of little wars with which England is for ever striving, yet amongst so large a number of troops illness is inevitable, and therefore hospitals and nurses must become permanent institutions. Some of the leading officials in the American army are much impressed with the Army Nurses' Training School at Netley, and consider that, with some modifications, the system of training pursued with the "Army Sisters" may, with advantage, be adopted across the Atlantic. Of course nothing is decided upon as yet, but the matter is receiving much attention amongst those who have witnessed the excellent work of the volunteer nurses under Miss Dorothea Dix. This lady has shown wonderful powers of organisation; and she and her staff have responded admirably to the calls made upon them, but the work they had to do and the work that must always remain to be done when there are wounded and sick soldiers to be nursed, only illustrates more vividly the need of a permanent staff of trained army nurses.

### LAYING THE FOUNDATION-STONE AT GARTLOCH.

If it be necessary for nurses who attend on those suffering only from physical ailments to be housed in a separate home, how much more is this essential for those who have the care of the mentally-afflicted. This was the keynote of all the speeches at the laying of the foundation-stone of the new nurses' homes at Gartloch Asylum, and it is one in which we heartily concur. The new home, when finished, will accommodate all the female nursing staff, which numbers about sixty. It will be built in three storeys, and each storey will have several separate bedrooms, a sitting-room, and a kitchen for the preparation of light refreshments, the regular meals being served, as hitherto, in the main buildings. On the ground floor are also the matron's and visitors' rooms, and the entrance is arranged to give access with equal facility both to the asylum and to the hospital. The quarters for the night nurses are approached by a separate staircase, and are situated on the top storey. The grounds behind the new building will be laid out for the nurses' recreation, and the cost of building and furnishing is estimated at £20,000. The exodus of the nurses will place between thirty and forty beds at the disposal of patients, of whom there are now 480.

### THE VICTORIAN ORDER OF NURSES.

The Hospital hears from its special correspondent in Klondyke that the Victorian Order of Nurses, both at Klondyke and also everywhere else, are doing splendidly, and that there are no complaints of interference.

### THE ESCUTCHEON OF THE GREEN CROSS.

The sign of the red cross has now for long been the symbol of succour on the field of battle. The originator of the idea and founder of the society is M. Henri Dunant, who resides in Switzerland. A few months ago he conceived the project of a new international society, this time for the befriending of women. For this organisation he has chosen the sign of a green cross, and Belgian ladies have been the first to put into practice his new precepts. The Belgian Society of the Green Cross is composed of three grades —patrons, who lend their names and influence to the work; committees, who bind themselves to give all possible help to persons asking their assistance; and auxiliary ladies, who make known the workings of the society, and introduce those desiring help to the helpers. These auxiliaries are often tradesfolk, who put up in a shop an escutcheon bearing a green cross, so that young girls and distressed women may through their agency be sent at once to the central committee. The organisation is receiving much attention in Holland, Russia, and Switzerland, and doubtless something more will soon be heard of it in England. The problem of banding women together in their own interests is a difficult one, but, if the obstacles can be overcome, the amount of good to be done by it is incalculable. Our nurses, too, must profit, and those in Belgium who are in trouble may claim help, without any qualification save that they are women and need it, wherever they see the sign of the green cross. Money is rarely given, but the assistance need not be less effectual for that reason.

### A CHILDREN'S HOME.

THE Children's Home is a useful little home, deserving of support, judging by the amount of work accomplished on a small income, and from the names of more than one business-like society appearing on its subscription list. But it needs more liberal support. It is situated at Much Hadham, Herts, and sixteen youngsters from the hospitals and slums of London are gathered within its hospitable walls, and allowed the freedom of the garden and surrounding fields until at least some measure of health and contentment is restored. Kindness, wholesome fare, and fresh air make a child's happiness. Alas! that gifts of which Nature is prodigal should be denied to her helpless little ones. Last year 119 cases were admitted, at a total (ordinary) expenditure of £429.

### A THOUGHTFUL GIFT.

A GIFT is valuable in proportion to the thought of the giver. The sisters, nurses, and staff of the Paddington Green Children's Hospital are happy, therefore, in receiving from Mr. J. Hawkins tickets of admission to the Alexandra Palace, Wood Green. The gardens will afford them many delightful hours in the open air, and the numerous entertainments will supply what so greatly adds to the enjoyment of a holiday—something to see, hear, and talk about.

### THREE YEARS' TRAINING FOR SOUTH AFRICAN NURSES.

THE period of training regarded as necessary for a nurse has lately been under discussion at Capetown, where hitherto a two years' course has been regarded as enough. Now, however, that there are plenty of candidates to train it is obvious that there is much to be gained in every way by increasing the time to three years. In the first place, the hospital lose their junior nurses just as their services are most valuable; and secondly, it is impossible to give the nurses themselves a first class training in the shorter time. This is the opinion expressed by Dr. Greathead at the Cape Medical Council, and with the result that the certificates granted by that body will for the future be only given after the longer term of probation. Another point was carried that sounds somewhat strange to English ears, namely, that the clause permitting private practitioners to train nurses should be eliminated from the regulations of the council. The consensus of opinion that three years is necessary to give the minimum training in nursing shows that the rule is founded on practical experience, and is therefore not a fad to be discarded with impunity.

### A SEVERE LESSON.

AN inmate of the Larne Workhouse helped herself to a bottle of poison from the nurse's store-closet and drank it. The medical officer applied the stomach pump, and the patient is progressing favourably. The nurse, who admitted that she had left the keys in the door whilst she was absent from the ward, has received a somewhat severe lesson against carelessness in small things.

### THE FIRST NURSE.

MISS ROSE PETTY, a Scotchwoman, trained at the London Hospital, is the first nurse appointed by the London School Nurses' Society. She will reside at the Hoxton Settlement, and pay weekly visits to the schools in the neighbourhood. Miss Petty also holds the certificate of the Sanitary Institute.

### HUMOURS OF BUMBLEDOM.

THE Guardians of Conway have more than ordinary difficulty in finding a nurse for the workhouse, because it is necessary that, in addition to her other qualifications, she should also be able to speak Welsh. One advertisement having proved fruitless, an amusing consultation took place between the members of the board, and the chairman suggested that it should be made known that a matrimonial agency had been established in the place as an inducement to candidates for the post. We much fear that even had it been practical to carry the jest into practice, the nurse attracted by the matrimonial agency would scarcely be an acquisition to the workhouse, and that the guardians would have found that the troubles incident to having no nurse were light in comparison to husband hunting persons in nurses' attire.

### SHORT ITEMS.

THE amount realised by the two days' bazaar at Tain in aid of the Nursing Association was £378.—The Health Department of the City of Buffalo, U.S.A., in 1890 decreed that it should be unlawful to make or sell any feeding-bottle or apparatus for feeding children in which rubber tubing was used. Medical authorities consider this a most desirable restriction, as many infantile complaints undoubtedly arise from certain kinds of feeding-bottles.—A second nurse is to be engaged by the Llandudno Nursing Association. The extraordinary feature of the annual report is that the expenditure has been only £100 and the income £262.—A copy of "an original water colour drawing, recently sketched in Denmark by the Princess of Wales," appears as a supplement to this week's *Gentlewoman.*—The Local Government Board have drawn the attention of the St. Saviour's Guardians, Southwark, to the bill for refreshments bought for the infirmary nurses' ball. The funny part of it is that the chairman knew nothing of the matter until it was thus brought to his notice. Exceptional expenditure ought certainly to be especially sanctioned by the guardians.—At a public meeting held at Roche, near Plymouth, it was unanimously resolved to appoint a nurse for the district. The rector's wife, Mrs. Thornton, is much interested in the matter, and numerous subscriptions have been promised.—Mrs. Sarah Moss, a midwife, practising in the neighbourhood of Spitalfields, being unable to collect her dues after rendering her services, adopted the plan of demanding payment in advance. This so enraged two of the residents in the vicinity of her home that they annoyed her by ringing her bell and calling her offensive names. The magistrate bound over both to keep the peace.—The secretary of the Hospital for Sick Children, Great Ormond Street, will gratefully acknowledge any contributions to the fund of £10,000 needed to complete the deposit of the purchase of the adjacent property, which will afford the much-needed accommodation for the nurses; £3,000 i still wanted.—Miss Ellen Court, matron of the Windsor Royal Dispensary and Infirmary, has been summarily dismissed, and her place temporarily filled until the appointment of a permanent officer.—Miss Hewlett, for many years the superintendent of the St. Catherine's Hospital, Amritsar, in the Punjaub, has in the press a volume of stories of missionary work amongst the blind in India.

### Notes on Gynæcological Nursing.

By Arthur E. Giles, M.D., B.Sc., F.R.C.S., Assistant Surgeon Chelsea Hospital for Women.

### III.—SOME ANATOMICAL CONSIDERATIONS.

My readers probably know something of the anatomy and physiology of the pelvic organs, but it will be convenient to refer to one or two points which bear more especially on nursing procedures. The "vulva" is the name given to the external parts and comprises the labia majora and minora, or greater and lesser lips; between them are seen, first the clitoris in front, then the orifice of the urethra, and immediately behind this the orifice of the vagina. Between the latter and the opening of the lower bowel (anus) is the perineum; this is the site of the operation of perineorrhaphy. The labia are sometimes the seat of cysts or abscesses, which may have to be dissected out or incised. In passing the catheter, by the touch alone, the opening of the vagina is taken as the guide, the patient lying on her back or on the left side. The forefinger of one hand is placed just within the entrance to the vagina, while with the other hand the catheter is guided along that finger, and so into the urethra. I do not, however, at all recommend the passing of a catheter in this way; it is done in order to spare the feelings of the patient, but unless it is done skilfully and gently it causes much more discomfort than if the nurse looks to see what she is doing; and it has the great disadvantage that it is the less cleanly method. It is, of course, most illogical to carefully sterilise the catheter and then introduce it without washing the vulva and urethral orifice; and so, in my opinion, it is much better for the nurse to first cleanse the urethral orifice and then introduce the catheter directly into the urethra, aided by the sense of sight, thereby avoiding the chance of the catheter becoming contaminated before it is passed.

The position and direction of the vagina are important in relation to the process of douching. The vaginal canal passes, not directly upwards, but upwards and backwards, and might be represented by a line passing from the orifice to the small of the back, at the top of the sacrum. This, therefore, is the direction in which the nozzle of the douche should be passed; if the patient is lying on her back, the tube must be made to point somewhat downwards towards the bed. Further, in introducing the tube, care must be taken not to press it forwards; this would compress the soft parts in front of the vagina up against the hard pubes, and would cause the patient pain; but it must be passed pressing rather back against the perineum—it will then be found to enter easily and painlessly. The total length of the vagina is 3½ in. to 4 in.; the nozzle should therefore be passed this far, in order that the douche may reach the upper end of the vagina; but it should not be passed further, lest it cause the patient pain. Another small point is important in this connection, and it is this: The labia must be gently separated with the fingers while the nozzle is being introduced, otherwise the pubic hairs may be caught in, and this would be unnecessarily painful. In giving an enema the nurse should remember that the rectum passes at first almost straight up in the middle line; but higher up it turns off backwards, and then to the left, as it passes up; and this direction is to be borne in mind when a longer tube is being introduced, such as a long rectal tube for the relief of flatus after operation.

### The Preparation of a Patient for Examination.

In many cases a patient is examined in bed, and should then be undressed in the ordinary way. In other cases the examination is conducted on a couch, either in the outpatients' room or in the surgeon's consulting room. The patient must then be undressed sufficiently to allow of the chest and abdomen being examined without impediment; if "combinations" are worn, they should be removed, and the petticoats and vest or bodice left on. In any case the corset should usually be removed. The position in which the patient is to be placed will depend partly on the surgeon's preference, but usually the patient should lie, in the first instance at least, flat on her back, to allow of the examination of the abdomen. For the vaginal examination one of three positions may be required. (1) The patient lies on her back, near the edge of the couch or bed by which the surgeon is standing, and with the leg on that side well drawn up and the other leg turned outwards. (2) The patient lies on her left side, with the knees drawn right up to her chest, the buttocks brought well over to the edge and the shoulders lying towards the middle of the bed; the left arm should be brought out behind the patient's back, so that her chest is almost flat on the bed. This is known as Sims' or the semiprone position. (3) The patient lies on her back with the buttocks drawn down to the foot of the couch, or to the edge of the bed, the patient in the latter case lying across the bed; the feet may be allowed to rest on two chairs, or on special supports for the purpose attached to the foot of the couch. Sometimes the thighs are bent up over the patient's abdomen, and either held by an assistant on each side, or secured by a special arrangement of straps, known as a "crutch." This is the "lithotomy" position; but it is used for vaginal operations or for an examination under an anæsthetic, rather than for a simple examination.

The preparation for examination includes the getting ready of various appliances, as follows: (1) Lubricants, according to the surgeon's preference, such as white vaseline, glycerine perchloride of the strength of 1 in 1,000, carbolised oil, thymol soap (a mixture of thymol soap and glycerine), or carbolised vaseline. It must be remembered that unless this last is carefully prepared the carbolic crystals may somewhat separate out from the vaseline, and there is then a risk of a drop of almost pure carbolic being brought in contact with the patient's skin—a most undesirable thing, since pure carbolic is a strong caustic. (2) Finger cloths, hot water, soap, and a clean towel. (3) Some absorbent cotton-wool in sponge-holders. (4) Certain instruments, viz, a sound, volsella, two or three Playfair's probes, dressed with cotton wool, a catheter, and some specula, such as Ferguson's, Sims' (the Duckbill), or Neugebauer's. These instruments will be referred to later in relation to the preparation of patients for operation. When opportunity allows, it is a great advantage, for the purposes of examination, that an enema should be administered beforehand, and the patient should empty the bladder, or the water should be drawn off with a catheter. I need hardly say that the catheter should not be used unless this is necessary.

### "THE HOSPITAL" CONVALESCENT FUND.

We have received several queries lately asking how to obtain assistance through this fund. A letter of application addressed to the secretary, at these offices, accompanied by one from a medical man, mentioning the nature of the illness from which the nurse has suffered, and stating the necessity for rest and change, and also a letter of recommendation from some responsible person, is all the formality necessary. We are glad of this opportunity to remind nurses who have been helped, and others, that our funds are low, and their efforts to increase them will be welcome.

### Antiseptics for Nurses.

By a Medical Woman.

### XVII.—CHLORINE AND ITS COMPOUNDS.

Chlorine was first discovered about 125 years ago, and is still a favourite and popular disinfectant, especially for drains and for disinfecting rooms after infectious diseases. It occurs in various forms, viz., chlorine gas, chlorine water, chlorinated lime, and chlorinated soda, together with their various solutions. All preparations of chlorine are antiseptic, disinfectant, and deodorant, and all are very easily prepared, and, consequently, cheap. Chlorine is a yellowish-green gas, having a very peculiar, penetrating, and disagreeable odour, even when it is only present in the most minute quantities, whilst in large quantities it is irritating to the eyes and throat, and may cause severe inflammation of the mucous membrane and even death when inhaled. Chlorine gas does not occur free in nature, but is obtained from the various chlorides, and especially sodium chloride or sea or rock salt, by treating it with strong sulphuric acid and manganese dioxide. These being heated together chlorine gas will be given off, and is purified by passing it through water and then collecting it. The gas is exceedingly soluble in water, and also combines readily with other substances, especially with lime. To form chloride of lime, all that is necessary is to pass chlorine gas into a chamber, of which the floor is covered with slaked lime two inches thick, and the gas is completely absorbed by the lime. Chlorine water is merely chlorine gas passed into water, which is subsequently shaken to ensure all the gas being absorbed, and thus is obtained a yellowish-green liquid with a strong smell of chlorine. To obtain chlorinated soda, solutions of chlorinated lime and carbonate of soda are mixed together, resulting in a colourless liquid having a feeble odour of chlorine and an astringent taste. Hydrochloric acid is obtained by the action on sodium chloride of sulphuric acid. When the two are heated together, a colourless gas is generated, heavier than air, and very soluble in water, and when water is saturated with it the ordinary hydrochloric acid, known commercially as muriatic acid, is produced, which is a colourless fluid with a pungent odour, is very acid, and fumes when exposed to the air; these fumes being very irritating to the mucous membrane of the throat and to the eyes. Hydrochloric acid may also be obtained when equal parts of chlorine gas and hydrogen are mixed and exposed to daylight; but the first method is the one employed usually to produce it. All these preparations owe their disinfectant powers to the presence of either chlorine or hypochlorous acid, the latter being given off abundantly, especially by chlorinated soda and chlorinated lime, and being also formed whenever free chlorine comes into contact with moisture. It is an actively oxidising agent, but is very unstable and gives up oxygen readily. Chlorine acts as a deodoriser in two ways—first, by destroying the germs which produce putrefaction, and, secondly, by destroying the noxious gases produced by decomposition, viz., ammonia, sulphuretted hydrogen, and sulphide of ammonium, which cause the disagreeable odour of a sick-room. Being in a gaseous state, and, therefore, able to diffuse through the air, chlorine is admirably suited for deodorisation, as it will penetrate into every corner of a room and destroy all noxious and offensive gases. It must be remembered, however, that the smell of chlorine gas hangs about a long time, and is very disagreeeble to many patients. When chlorine gas is used as a disinfectant in such a diluted form that the air is still respirable, its disinfectant action is probably *nil*, and it is very doubtful whether chlorine in a gaseous state can destroy germs and their spores, although many infectious substances can be made by its means to lose their power of spreading disease. To disinfect an unoccupied room after infectious disease the air must be thoroughly impregnated with chlorine to the extent of one volume of chlorine to every 100 of air. Koch declares that chlorine is superior to sulphurous acid for the purpose of fumigating if it is used sufficiently strong and concentrated; and says that for this purpose it is necessary to take not less than 15 lb. chloride of lime and 22 lb. hydrochloric acid for every 1,000 cubic feet of air space in the room. This is, however, a much larger quantity than is considered necessary in this country, where authorities differ somewhat in the amounts recommended, which vary from 1 lb. of chloride of lime and two pints of hydrochloric acid to double those quantities. Whatever quantities are adopted, the chloride of lime should be distributed in several glazed earthenware vessels placed in different parts of the room, and as high as possible, owing to the heaviness of chlorine gas, which might otherwise form a heavy layer on the floor of the room and not reach the upper parts in a concentrated form. When all openings have been carefully closed and the windows pasted up, and every preparation made for the operator to leave the room at once, the due quantity of hydrochloric acid should be poured into each vessel containing the chloride of lime and the door pasted up and the room kept closed for twenty-four hours and then thoroughly ventilated. Some authorities advocate the preparation of chlorine gas by placing in an open dish 8 oz. common salt, 2 oz. manganese dioxide, and a mixture of 2 oz. each of water and sulphuric acid. The drawback to disinfection by chlorine gas is that many kinds of materials are greatly damaged by it. Dr. Rideal says of disinfection by chlorine gas or its compounds that, although it has always maintained a certain degree of favour on account of its cheapness and efficacy, still the disagreeable odour and the corrosive action of the whole class of chlorides have caused it to be very strongly objected to, and many people will not use it for these reasons. The cause of the general dislike to chloride of lime is not far to seek, apart from the odour, which to many people is singularly disagreeable. Its cheapness had caused it to be thrown in shovelfuls on iron gratings, which it rusted, or down the metal syphons of water closets, which, whether lead or iron, it often pierced, and its lumpy character produced by its deliquescent nature often led it to be washed away without dissolving, and in consequence its disinfectant effects were very imperfect. Disinfection carelessly or insufficiently done is worse than no disinfection at all, as it conveys a sense of false security. The fact that chloride of lime requires to be well stirred with water before being used, and that it deposits about 40 per cent. of insoluble matter, chiefly lime, has led to the re-introduction of hypochlorite of soda, which gives no such insoluble deposit. Each of these substances is useful in its way, and hypochlorite of soda is sold in solution under the name of "chloros," which is said to contain 10 per cent. available chlorine, and the solution is directed to be diluted with 100 parts water.

Euchlorine is a mixture of chlorous acid and free chlorine. It is obtained by gently heating together, by putting them in a receptacle placed in warm water, strong hydrochloric acid and chlorate of potash. It may be used for disinfecting rooms instead of chlorine gas, the great advantage of euchlorine being that it has a more agreeable odour and is more easily obtained, but it is probable that it is inferior to pure chlorine in disinfecting powers.

Chlor-alum is a weak solution of chloride of aluminium, obtained by heating alumina and charcoal in a current of chlorine gas. It is a weak disinfectant, as the amount of chlorine gas which supplies its disinfecting properties is not great, and it must be used in large quantities; but it is very cheap, and is said to be non-poisonous. In sufficiently large quantities it is said to have proved an efficient disinfectant.

Hypochlorous acid is obtained by passing a current of chlorine gas through water, in which is suspended recently precipitated calcium carbonate.

### How to Start a County Nursing Association.

By LADY BAKER.

HAVING been hon. secretary for over five years of the first nursing association started on purely county lines, viz., with general, executive, and medical committees, composed of some of the best known names in the county, perhaps a few hints on the above subject may not be unwelcome to the general public, who, in many cases, are now proposing to work on the same lines. When I first propounded the notion in my own county, all my friends, with but one or two exceptions, endeavoured to persuade me to give up such a bold and impossible idea. "Content yourself with your own neighbourhood," said one (I confess I frequently, in moments of despair, wished I had). "Start a nurse for your own village," said another (the parish was manifestly too small, but in trying to do so I bought some experience). "Everyone hates organisation," said a third. This I acknowledged to be a fact—organisation, though necessary, is not popular, especially with a philanthropic spasmodist, if I may coin a word. However, a few fanatics among us drew up an ideal plan of action, as it seemed to us, ranged ourselves in the forefront of the battle, with some confiding friends in the rear, and set to work.

Of the apathy of the public bodies, with whom we broke many a lance in defence of the laws of health; of the cutting remarks hurled at us by insanitary landlords; of the reluctance of the pauper to face the nurse's unwelcome soap; of the doubtful smiles of time-honoured medical men; of that most disheartening cry of all, that of the dogged rustic, "We don't want no new-fangled nurses!"—over these I will draw a veil, lest those who would follow our example should find their hearts fail them, as mine would have done had I foreseen what fate had in store for me. But "dogged does it" —fight even the rustic with his own weapons, and at last he will yield with a smile, when the grip of grim pain is upon him, to the gentle hand of a ministering woman.

It will, however, be of more use to proceed at once to give the results of a somewhat checkered experience than to dilate on minor difficulties, so I will forthwith endeavour to answer the oft-repeated question, "How to begin?"

The first step to take in starting a county organisation is to enlist the sympathy of some of the most influential people in the county, *i.e.*, those who can bring into public use not only money and influence, but brains and good business heads, and, above all, leisure. Great names are invaluable, especially when backed by great cheques! but time and work can seldom be expected of them; they are, as a rule, too fully occupied with other matters to attend committee meetings, or come long distances to discuss minor details.

Having, however, secured the promise, not only of their names, but of practical support in some form or other, even if only a promise to preside at an annual meeting (as without some practical support a name, however great, is of little real value in these democratic days), forthwith proceed to make up your executive of those who are actually engaged in doing nursing work in some form or other in the county. Among them have a sprinkling of hard-headed business men or women, who will keep the superabundantly sympathetic element in check, and watch the expenditure of every sixpence as it is wrung from a sometimes reluctant public.

Above all things, do not neglect those who have been at work, though possibly not on the same lines, before you. This is a lesson which all of us who take up a work new to ourselves are too apt to overlook. Until we seek them out we have no idea how often other humble and unseen workers have each, in their own little limited area, been preparing the ground for us. Let them have a share of the credit. It is this ignoring of little previous attempts, even though they have been unsuccessful ones, which makes a great enterprise so slow to advance. It arouses so many little jealousies by saying, "I came, I saw, I conquered." "We were already there," say the humble beginners; "we, too, saw, and our work helped you to conquer."

Draw them in from the very beginning, from the humble cottage nurse, managed by the parson's wife, with her little professional knowledge, but her warm sympathy, to the highly-trained district nurse of the Lady Bountiful. Let their work—much or little—be fairly represented on your new executive committee, and let the voice of their experience, even though somewhat one-sided, be heard from the very beginning of the new enterprise. This will, I grant, in some cases be the most difficult part of the work.

The Lady Bountiful so much prefers paying for her own nurse, and managing her entirely herself, much as she would her own dairy. Why should a committee composed, say, of the parson's and farmers' wives, the heartless parish doctor, or that tiresome sanitary inspector, have any right to criticise her or her nurse, and suggest, say, that the dear old woman down at the lodge is suffering, not from rheumatism, but from drains, or the brandy bottle, when she herself is sure that all the poor old thing wants is a little port wine and good nursing? Why should the poor dear people be asked to give their hardly-earned pennies when she can give her pounds, that is to say, as long as she feels inclined to? What becomes of the nurse, and the poor people, when she is on the Continent, or up in town for the season, or in Scotland, or still more, is gathered to her forefathers, she does not wait to inquire. Nor why the "penny check" should have stopped malingering and hysterias, and wasting the nurse's time so effectually in the next village. Let her be. After a while our Lady Bountiful will ask, Why the next club, with its committee and its regular rules, seems to keep their nurse so much longer than she does? And how the nurse manages to collect so many pennies in the course of the year from the club members? and a few other pertinent questions. And you will find in a little while that she has co-opted a few neighbours while she is away, and those few friends will co-opt a few others, and so formed a committee, and that she has come round after all to the "benefit system," and before long she will have caught up the new committees, with their regular rules, their quarterly meetings, their club payments, their recognised members, and all the rest of it, and so a little leaven will leaven the whole lump.

Other difficulties you will have to outlive, too, in getting already established town districts to join a new organisation. They do very well as they are; they have a committee (managed by the parson's wife, as is also the nurse); what does it matter if they are all Church people on the committee, and the Dissenters don't get nursed? They never heard of a sick Dissenter applying for a nurse; of course not, they are not represented on a purely parish club. The very idea of talking of a public town nurse, or asking the mayor to raise funds or preside at the annual meeting! why, is not he a Wesleyan himself? In vain you plead "the quality" of nursing, like that "of mercy, is not strained," and that a sieve which excludes Jews, Turks, infidels, and heretics, is not needed in the presence of our grim master, Pain. Let not even the parson's wife ruffle your or her serenity; let her be. When the mayor makes a handsome donation to the funds of the district nurse on the condition of joining the county association, she will find out that after all the local rules are not incapable of a little relaxation, and that she can do no less than ask the mayor's wife to come on the committee, and in a little while her lump will also be leavened. Thus, after a while, she, too, will take her place, as representative of her district, side by side with the Lady Bountiful, and they will compare notes, and the town experience of the one will modify the village experience of the other. Presently the strong common sense, and excellent business capacity of the mayor's wife—Dissenter though she be—will rouse the genuine surprise and admiration of both, to the closer drawing together of the bonds of Christian charity. Thus, your executive committee, with its originally conflicting elements, will gradually work up into a compact and harmonious whole. Once a year you will be able to gather all your friends and supporters together at a general committee, to compare notes and report progress, and you will find that, after all, if you are county secretary, you have a good deal to put into your yearly report; and you will remember that unity is strength, while to have been the means of giving even one extra cup of cold water in the name of a disciple to some poor dying soul is a thing to be thankful for, and you will take heart in the thought that you have made even a beginning.

Of the medical committee and its pitfalls, of local committees and their tiffs, of the patients and their vagaries, of the cottage nurses and their wrongs, of the trained nurses and their rights, I must crave your patience if I treat of them in a future number.

### A Walk with a Weinyddes (Nurse) of Wild Wales.

"IT'S always raining at Blaenau Festiniog," was her consoling observation upon the downpour which completely hid the rugged heights of Moelwyn. "It must be the slates, I think. Get through the tunnel, and there's fine weather on the other side of the mountains!"

A rival to Harley Street in length, the quarrymen's town winds its way by ins and outs and ups and downs (with occasional side excursions), as one main street round the amphitheatre of hills forming the head of Festiniog Vale, from Tanygrisiau to Llan Festiniog. A matter of some four miles of walking for the Queen's Nurses, who are a new institution here, the "Cymdeithas Gweinyddes au Festiniog" (or Festiniog Nursing Association), having been only founded this year. The people had an idea before they came, that a district nurse was a kind of domestic arrangement inspector; every house in the village was cleaned up in anticipation of their arrival, and great was the disappointment in healthy households when the new inspector failed to visit them!

About five o'clock on a clear afternoon, hundreds of probable patients may be seen rapidly descending to their homes from the slate quarries on the mountains; they come crouched on curious little trollies, which they roll for themselves down the truck lines. Bicycle coasting seems tame sport after one has watched this exciting process. Naturally a great part of the Queen's nurse's work is surgical, but a glance at her case book tends to confirm the impression obtained by observation of the people themselves that, while the men and children are exceptionally healthy and intelligent, the women are anæmic and delicate. Here in the heart of wild Wales English is an exceedingly limited commodity. "If I were to speak to my patients in English," nurse remarked, "they would say at once, 'How proud she is.'" So only Welsh women are available for the work in this district. The quarrymen earn from £5 to £7 a month, she further informed us, and there is very little real poverty in Bleanau. Though the ancient costume is discarded, there is a substantial quality about Sunday clothes which contrasts favourably with the flimsy finery worn by working women in London or our large towns, and there is nothing suggestive of cheapness even in a quarryman's labouring dress.

The twelfth century gossiping Geraldus of Wales had stories to tell about Welsh part-singing, and certainly the men of Bleanau Festiniog have inherited the musical talent of their ancestors. Standing outside a "Bethesda" or "Jerusalem" on Sunday evening the harmonious, unaccompanied part-singing recalls the beautifully pathetic psalm singing during the Holy Week offices in Italian churches; possibly the tunes are very ancient. The Bleanau Festiniog choir recently had the honour of singing before the Queen, and in a poor cottage at Llan Festiniog dwells a bard whom gold cannot tempt to part with the chairs he won for his poems at the Eistedffods.

### Royal National Pension Fund for Nurses.

A NEW prospectus of the Pension Fund has just been issued by the Council. It forms a neat little volume. The new edition is very much clearer and more lucid than the last, the chief points being indicated by difference both in type and colour. Sickness and accident assurance is fully dealt with, and all the "special regulations" which the Council have found necessary to draw up are printed in full. The tables have been completely remodelled; instead of having all commencing ages on one page and in small and obscure type, each commencing age is now dealt with on a separate page; the amount of premiums requisite to assure pensions of £7 10s., £10, £15, £22 10s., and £30 being indicated. For the first time sickness assurance rates are shown separately, so that a nurse may see at a glance the exact amount requisite. The old Table "F" (combining pension and sickness assurance rates) has now been changed to Table "S," and is merely a combination of the pension and sickness assurance rates given in one line for the sake of convenience. Opposite every table is an explanation with examples showing how combinations of payments can be made to suit nurses who are able to pay in a lump sum in order to supplement the amount produced by a monthly premium. Single payment rates are given with full explanations. The amount to be distributed as a bonus for the five years ending December 31st, 1897, is given. In this connection the secretary desires us to state that the supplementary report promised in the last annual report will in due course reach the hands of policy holders, and that after that report is issued any policy holder whose policy has been in force at least five years will be informed of the amount of bonus addition made to her policy on application to the office. No doubt many nurses, already members of the Fund, will desire to possess a copy of the new prospectus; but as the postage is a very heavy item in the expenditure of the Pension Fund, we feel sure all who write will send a penny stamp to defray the cost of supplying it.

### Minor Appointments.

THE UNION WORKHOUSE, GRAVESEND.—Miss Lois Lightwater has been appointed Superintendent-Nurse of the above institution. She was trained at the Union Infirmary, Sheffield, where she afterwards acted as charge nurse and later as midwife. She worked as a private nurse at the Huddersfield and Halifax Nursing Home, and later took the post of charge nurse at the Toxteth Infirmary, Liverpool, and finally became night superintendent there.

CARDIFF UNION WORKHOUSE HOSPITAL. — Miss Mary Ann Camamite, who was trained at the Central Sick Asylum, Cleveland Street, London, was appointed Charge Nurse of the above on the 10th inst. She has previously held positions at the Home for Incurables, Leamington, and at the Northern Fever Hospital, Winchmore Hill, under the Metropolitan Asylums Board.

CITY OF COVENTRY FEVER HOSPITAL.—Miss Lilian King, who was trained at the Royal Infirmary, Blackburn, was elected Sister here on September 2nd, 1898. Her previous appointments have been charge nurse at her training school, and afterwards charge nurse, under the Metropolitan Asylums Board, at the hospital ships, and at Fountain Hospital, Tooting.

NEW MIDDLEWARD HOSPITAL FOR INFECTIOUS DISEASES, NEAR MOTHERWELL, LANARKSHIRE.—Miss Georgina K. Adams, who was trained at the Western Infirmary, Glasgow, was appointed Matron of this hospital on the 7th inst. She has previously been matron of the Forfar Infirmary.

CARNARVONSHIRE AND ANGLESEA INFIRMARY, BANGOR.—Miss Alice Hulme has been appointed Charge Nurse at the above institution. She was trained at the General Infirmary, Chester, and worked on the private staff of the infirmary eighteen months.

THETFORD COTTAGE HOSPITAL.—Miss Scully has been appointed Matron of this cottage hospital. She was trained at the North Riding Infirmary, Middlesborough, and continued to work at that institution.

### Training in the Provinces.

(*Continued from page* 205.)

### GLASGOW ROYAL INFIRMARY (582 Beds.)

### TERMS OF TRAINING.

The Glasgow Royal Infirmary was the first amongst the nurse-training schools of this country to adopt a system of preliminary training for its probationers, a distinction which it now shares with the London Hospital, where, however, the experiment is being tried on somewhat different lines.

At Glasgow the "preliminary education" required of candidates for training in the wards occupies a period of three months, during which time the pupil provides board and lodging at her own expense, attending two courses of lectures, examinations being held at the conclusion of each course. The first course, for which the pupil pays a fee of £2 2s., consists of twelve lectures and demonstrations on anatomy, and the same number on physiology and on hygiene. Only those who satisfy the examiners are eligible for further instruction. Those who pass attend a second course, consisting of twenty lectures and demonstrations on surgical cases, twenty on medical cases, and twenty practical lectures by the matron and housekeeper on ward work and cookery. For this second course the fee is £3 3s. Candidates are not eligible for these courses of instruction until they have satisfied the examiners as to their knowledge of grammar, composition, spelling, dictation, reading, writing, and arithmetic. The Leaving Certificate of the Scottish Education Department and the Senior and Junior Certificates of the University Royal Examinations are accepted as sufficient evidence of preliminary education. The age considered desirable for candidates is between 20 and 30 years.

The three months' instruction satisfactorily completed, the candidate enters the infirmary for three years' training at a salary of £12 for the first year and £20 the second, with board, lodging, and washing. At the end of three years the probationers may present themselves for a final and practical examination, on passing which they receive certificates as trained nurses.

All probationers are admitted upon the same terms, and are alike eligible for promotion after their training is completed, according to ability. Salary after the second year rises £2 annually to a maximum of £30 per annum. Laundry and indoor uniform are provided by the hospital.

### HOURS ON AND OFF DUTY.

The hours on duty of day probationers at the Glasgow Royal Infirmary are somewhat unusual. Probationers rise at 4 a.m., going into the wards at 4.30, when they are there provided with an early breakfast (their second breakfast they have at 7). They are on duty, allowing time for meals, till 4.30 p.m., when they leave the wards for recreation before supper and bed, to which they retire at 7.30 p.m. Assistant nurses on day duty are in the wards between 7.30 a.m. and 8.30 p.m., with meal times and two hours off in the afternoon from 4 to 6. Staff nurses' hours on duty are from 8 a.m. to 10 p.m., with two hours in the afternoon for recreation, and time for meals.

Assistant nurses take night and day duty alternately in periods of three months each. When on night duty they are in their wards from 9.45 p.m. to 10 a.m., deducting an hour for breakfast from 7.30 to 8.30 a.m. The return of the night nurses to the wards after this meal is arranged to allow them to see the doctors, with the view of keeping up their interest in their work. In practice the nurses are frequently free to leave the ward again before ten.

One day each month is allowed off duty, when nurses do not enter the wards at all. An evening pass is also given once a week when wished for. On Sundays the nurses are off alternately from 10 to 2 or from 2 to 10. The day off can be arranged for from 6 p.m. the previous evening when desired. Annual holidays are three weeks for probationers, and one month after three years' service.

Probationers have no class work after they begin their ward training, their theoretical knowledge having been already tested by examination before entrance into the hospital. This fact, of course, means that the strain on the nurses generally during their three years is very much less than when the theoretical and practical instruction is proceeding side by side, and that there is no enforced encroachment upon the stated hours for rest and recreation.

### MEALS.

It is a little surprising to find that the practice of allowing the nurses to take certain meals in the wards or ward kitchens still obtains at the Glasgow Royal Infirmary. The day probationers, who go on duty at 4.30 a.m., have "early breakfast in the wards" at that hour. The time table also says that "all nurses are allowed tea in the wards," and both lunch and afternoon tea are served either in the ward or ward kitchen. Day probationers have their second breakfast at 7 a.m., they lunch with the staff nurses between 10 and 11 a.m., dine at 2, and have supper at 6.45. The day assistant nurses have breakfast at 7, dinner at 12 30, and supper at 8.30; staff nurses breakfast at 7.30, have dinner at 2, and supper at 8 p.m. Night nurses have supper at 9.15 p.m., breakfast at 7.30 a.m., and dinner at 12.30 p.m. Allowances are given to the nurses for night meals, afternoon tea, and for "special duty."

### GLASGOW WESTERN INFIRMARY (400 Beds).

### TERMS OF TRAINING.

Candidates for training at the Glasgow Western Infirmary must be between 22 and 32 years of age, a trial of three months being exacted before applicants are accepted as probationers, when they sign an agreement to remain for four years in the service of the infirmary. No premium is required, probationers' salaries beginning for the first year at £10, and increasing to £15 for the second and third years. Staff nurses' salaries range from £25 to £30; sisters', from £30 to £40. Probationers leaving the hospital before the expiration of the term agreed upon may be required to refund part of their payments. Laundry is provided by the hospital, and indoor uniform.

Practical and theoretical instruction is given during the course of training by the sisters and by members of the medical and surgical staff, and certificates are awarded at the completion of three years' training, after passing a written and practical examination.

All probationers are alike eligible for promotion to higher appointments in the hospital, the test being one solely of merit and capacity, after the three years are finished. Night duty is taken alternately for a month or so at a time.

### HOURS ON AND OFF DUTY.

Nurses and probationers go on duty in the morning at 7, and leave their wards in the evening at 8 o'clock. Sisters go on duty an hour later in the morning. From these hours on duty must be deducted time for lunch, dinner, tea, and supper, and on alternate days three hours off for recreation. Night nurses are on duty from 9 p.m. to 8.30 a.m., returning to their wards after dinner to give in their reports. They are then off duty till they retire to bed at 12.30. One whole day, on which nurses do not go on duty at all, is given once a month. The annual holiday for probationers is about sixteen days. Sisters and certificated nurses have one month's annual leave of absence.

### MEALS.

The day nurses' breakfast is served at 6.30 a.m., lunch at 9.30, dinner at 12.30 and 1 p.m., tea from 4 to 5, and supper from 7 to 8 p.m. All meals are served in the nurses' dining-room. Allowances of butter, tea, sugar, &c., are made to the night nurses, and to day nurses for the morning lunch. Night nurses breakfast at 8.30 p.m., and dine at 9 a.m.

### For Reading to the Sick.

SANCTIFICATION.

"THIS is the will of God: even your sanctification."—1 *Thess.* iv. 3.

**Verses.**

Lord, if Thou Thy grace impart,
Poor in spirit, meek in heart,
I shall, as my Master, be
Rooted in humility.

Simple, teachable, and mild,
Chang'd into a little child;
Pleas'd with all the Lord provides;
Wean'd from all the world besides.

Father, fix my soul on Thee!
Ev'ry evil let me flee;
Always happy in Thy love,
Looking for my rest above.

Oh, that all may seek and find
Ev'ry good in Jesus joined.
Him let Israel still adore,
Trust Him, praise Him, evermore!

Jesus calls us from the worship
Of the vain world's golden store,
From each idol that would keep us,
Saying, "Christian, love Me more."
—*C. F. Alexander.*

Thus, dishonouring not her station,
Would my life present to Thee,
Gracious God, the pure oblation
Of divine tranquility. —*Wordsworth.*

The man who consecrates his hours
By vig'rous effort, and an honest aim,
At once he draws the sting of life and death.
—*Young.*

**Reading.**

It is a hard thing to give up our own will; it seems so right; it has been so well considered; it will prevent so much harm; it will do so much good! No wonder we try to get it; and that, when met by contrary winds, a storm ariseth! Nature and grace not only speak different languages, as we have seen, but they draw different pictures. The subject may be the same—the storm subsiding into a calm. Nature will represent the elemental strife, the waves lashed to fury, the vessels tossing to and fro; followed by the hushing of the winds, the scattering of the clouds, the breaking of the sun. . . . But grace will show unruly passions assuaged, thoughts brought into subjection, words that might do hurt hushed, the head bowed, the will subdued, and the image of God reflected by man. This is the more beautiful picture, and grace is the more wonderful painter! Whatever hindereth being taken out of the way, God's will is then ours; and "this is the will of God, even our sanctification." This sanctification involves purity of heart, conversation as it becometh the Gospel, putting our little feet into Christ's great footprints, love to the Brethren, prayer for the whole household of faith, doing good, ripening for Heaven. First, then, be ready to give up thine own will. Then, calm and tranquil, endeavour to learn God's will. Then be assured that it leads, though perhaps by a way you thought not of, to the one great end of sanctification.—*J. B.*

### Presentations.

MISS DOW, matron of the Middle Ward Hospital, Motherwell, has, on the occasion of her marriage, been presented by the staff with a silver salver; also a silver tea and coffee service, suitably inscribed. Miss Dow carries with her the sincerest wishes of all for her future happiness.

### Notes and Queries.

**The contents of the Editor's Letter-box have now reached such unwieldy proportions that it has become necessary to establish a hard and fast rule regarding Answers to Correspondents. In future, all questions requiring replies will continue to be answered in this column without any fee. If an answer is required by letter, a fee of half-a-crown must be enclosed with the note containing the enquiry. We are always pleased to help our numerous correspondents to the fullest extent, and we can trust them to sympathise in the overwhelming amount of writing which makes the new rules a necessity.**

**Every communication must be accompanied by the writer's name and address, otherwise it will receive no attention.**

*Milk Tabloids.*

(233) Can you tell me where I can obtain milk tabloids, which, when put into water or a cup of tea, will melt and take the place of ordinary liquid milk? I have tried Burroughs and Welcome's.—*Nurse Constance.*

We do not know of any such preparation.

*Parquet Floor Polishing Brushes.*

(234) What other firm besides Kent and Sons supply parquet floor polishing brushes?—*Matron.*

All large stores supply brushes for polishing parquet.

*Lying-In Homes.*

(235) Will "Jim" send name and address in accordance with our rule?

*Ladies v. Gentlewomen.*

(236) Will the paragraph on "Ladies and Gentlewomen" in THE HOSPITAL of last week have any effect in stopping domestic servants from becoming nurses?—*Agatha Jones.*

Not the slightest. Many matrons prefer the right kind of domestic servant to train, and many patients prefer such to nurse them.

*Nursing in India.*

(237) Kindly tell me what to do and to whom to write in order to obtain nursing in India. Is there a good opening? Is it better to join an institution?—*Warner.*

It is certainly best to join an institution until a good connection is established. The Up-country Nursing Association for Europeans, of which the Hon. Secretary is Major-General J. Bonus, The Cedars, Strawberry Hill, seems most suited to your requirements. The engagement is for five years, and the salary is Rs.75 a month, with board, lodging, and attendance.

*Dental Hospital.*

(238) Kindly tell me how one can get treatment at the Dental Hospital. If a letter is needed, and how to get one?—*L. R.*

No ticket is required for ordinary extraction at the Dental Hospital for London, 40, Leicester Square, W.C. Apply for full particulars before noon at the hospital, or send a stamped and addressed envelope.

*Post Graduate Training.*

(239) 1. Is a fee necessary in order to have alterations of name and appointments inserted in Burdett's "Official Nursing Directory"? 2. Does the East End Mothers' Home train for the L.O.S., and is the training as good as Queen Charlotte's for my purpose? 3. Is there a fever hospital where I can gain a certificate after a few months' training? I hold a three-year certificate from a general hospital, but wish to get a good all-round training to fit me for an institutional post.—*A. C.*

The Editor of Burdett's "Official Nursing Directory" is pleased to receive accurate and early information of all nursing appointments. This is inserted free of charge in the next issued edition of the work. 2. As you wish for a post in an institution the larger training school, *i.e.*, Queen Charlotte's Hospital, will suit your purpose best, as besides the training for midwifery you will also learn something of the management and organisation of the hospital, which will prove of value. 3. A six months' or longer course at a fever and other special hospitals will give you the experience you need, and for which you will require no certificate but the testimonial of your superior.

*Dispensing.*

(240) I want to learn dispensing, and do not quite know how to set about it. Can you tell me where are to be found the best places to learn quickly, where to get certificate, and when qualified is there any difficulty of obtaining post as lady dispenser?—*P. W. P.*

(241) I am anxious to qualify as dispenser. I have had over two years in practical experience. (2) Is it difficult to obtain a post when qualified, and (3) what is the average salary?—*Miss Haines, Lunatic Asylum, Cork.*

Either the "Certificate of Qualification to act as Assistant in Compounding and Dispensing Medicines" of the Apothecaries Hall, Blackfriars, E.C., or better still the higher qualification given by the Pharmaceutical Society, 17, Bloomsbury Square, W.C., is necessary. The school conducted by the Council of the Pharmaceutical Society is open to lady students, and there are private teachers. See "How to Become a Dispenser," Mirror, for April 3rd, 1897. 2. Not very. 3. From £70 to £120 at large hospitals.

*Children's Hospitals.*

(242) Kindly let me know the best children's hospitals for probationers?—*N. P.*

As about ninety children's hospitals are noticed in Burdett's "Hospitals and Charities," the question is wide. The three largest in London are, of course, the Hospital for Sick Children in Great Ormond Street, the East London Hospital for Children at Shadwell, and the Evelina Hospital for Sick Children, Southwark Bridge Road, S.E.

